# Development of a shallow-water underwater acoustic recording system — version 2

**DOI:** 10.1016/j.mex.2026.104112

**Published:** 2026-08-22

**Authors:** Donatas Bagočius, Aleksas Narščius, Gintaras Kučinskas, Gediminas Uskovas

**Affiliations:** a Klaipėda Higher Education Institution (KVK), Jaunystės str. 1, Klaipėda, LT-91274, Lithuania; b Klaipėda University, H.Manto str. 84, Klaipėda, LT-92294, Lithuania; c Lithuanian Maritime Academy of Vilnius Gediminas Technical University, I.Kanto str. 7, Klaipėda, LT-92123, Lithuania

**Keywords:** Environmental pollution, Underwater noise, Acoustic recording system, Hydrophone

## Abstract

Commercial underwater noise recorders are expensive and often vulnerable to loss or entanglement in fishing nets, while developing affordable alternatives is challenging and requires expertise in electronics and low-level programming. Consumer-grade microcomputer platforms offer a cost-effective and efficient solution for monitoring underwater noise, particularly in shallow coastal areas. This paper investigates a method for developing an autonomous underwater acoustic recorder based on earlier work. The developed system able to store acoustic data over mid-term deployments and is suitable for applications such as environmental noise assessment, aquatic animal monitoring, and recording impulsive or vessel-generated noise. The performance of the developed device was validated in air using a purpose-built acoustic coupler and sine wave signals, following a comparative hydrophone system calibration procedure and tested during the field measurements in the Klaipėda Harbour area, Lithuania.

• Development of a low-cost autonomous underwater sound recording system.

• Validation of the system using a comparative hydrophone calibration procedure.

• Experimental measurements of ship noise using the developed system.

## Graphical abstract

**Figure:**
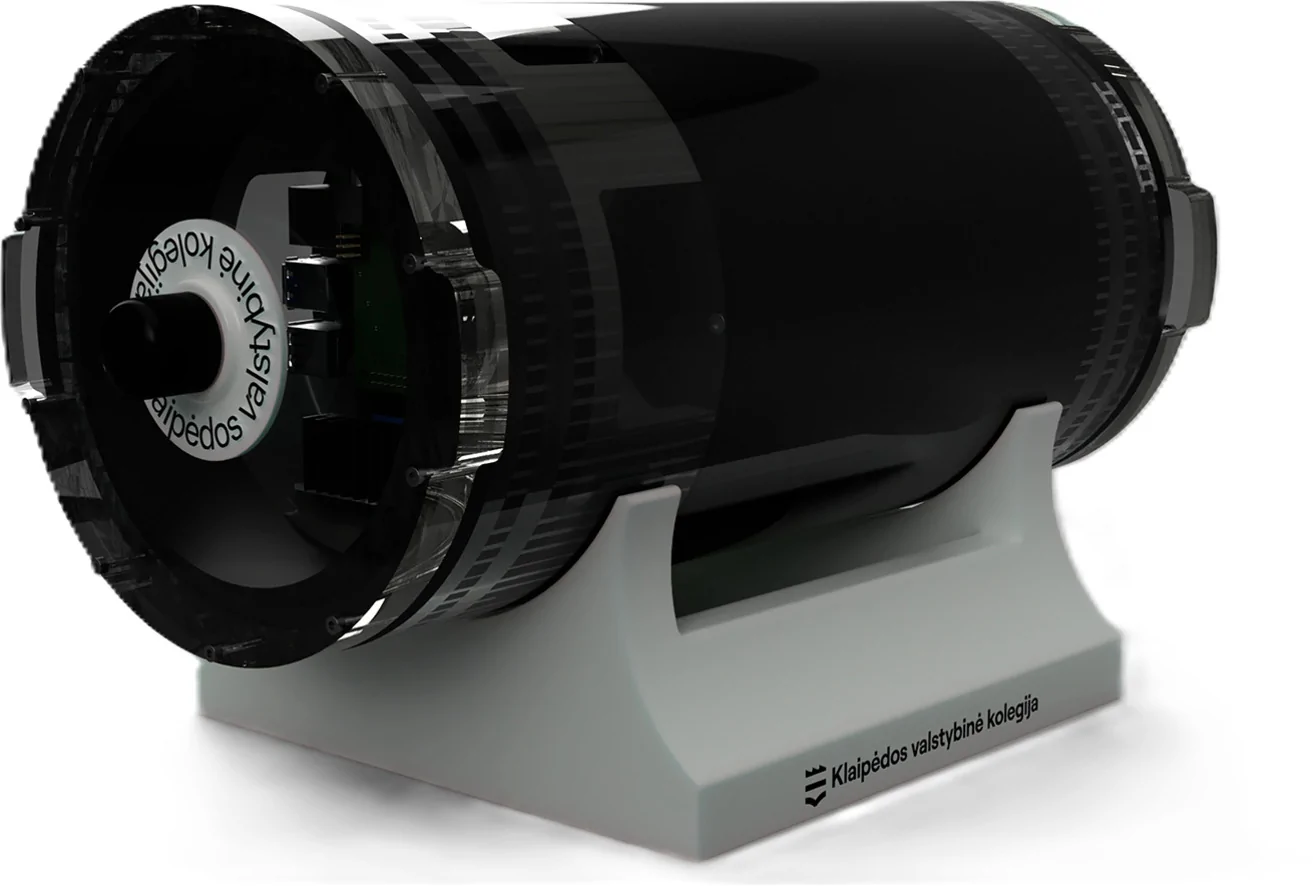

## Specifications table

**Table:** 

**Subject area**	Environmental Science
**More specific subject area**	*Underwater noise pollution*
**Name of your method**	*Development of a Shallow-Water Underwater Acoustic Recording System*
**Name and reference of original method**	*Au, W. W., & Hastings, M. C. (2008). Principles of marine bioacoustics (Vol. 510). New York: Springer* *Caldas-Morgan, M., Alvarez-Rosario, A., & Rodrigues Padovese,* L. *(2015). An autonomous underwater recorder based on a single board computer. PloS one, 10(6), e0130297*
**Resource availability**	*Supply list (cost):* *1. Aquarian Audio H2D(M) Hydrophone (€250.00)* *2. Raspberry Pi 5 Microcomputer (€90.00)* *3. HiFiBerry DAC+ ADC Pro Shield for Raspberry Pi (€70.00)* *4. OEM DC/DC Step-Down Converter, 5–40* *V to 1.2–35* *V (€8.70)* *5. microSD Card, 125 GB (€30.00)* *6. 3.5* *mm Audio Jack Connector (€8.60)* *7. USB-C Connector (€7.90)* *8. Low-Power Cable, 0.125″ (€3.10)* *9. Audio Cable, 0.125″ (€14.60)* *10. Alloy Wire Terminal Connectors, 2 pcs (€3.00)* 11. 2-Pin Battery Connector/Splitter (€7.20) *12. LiFePO4 Rechargeable Batteries, 60 Ah, 3.2* *V, 2 pcs (€112.80)* *13. 3D Printing Materials (€50.00)* *14. Industrial Potting Epoxy (€16.50)* *15. M0.5 Screws (€2.00)* *16. M5 Screws (€2.00)* *17. Cable Ties (€1.00)* *18. Blue Robotics Watertight Enclosure, 6″ Series (€546.00)* *19. Blue Robotics Pressure Vent (€10.00)* *20. CNC Aluminum Extrusion Profiles 2020, 500* *mm Length, 8 pcs, Including Connectors (€71.30)* *21. Marine Plywood, 9* *mm Thickness, 205* *×* *205* *mm, 2 pcs (€20.00)* *22. Miniature Loudspeaker G402 (€14.60)* *Total cost (excluding VAT and shipping): €1339.30*

## Background

Man-made activities introduce several forms of anthropogenic energy into the marine environment, including acoustic, light, electromagnetic, thermal, and radioactive energy. Of these, underwater acoustic energy is the most widespread and pervasive [1]. These activities are adding the anthropogenic underwater noise to the Seas, while concerns are given to possible effects of underwater noise interference with marine fauna communication, environmental sensing, acoustic and non-acoustic behaviors, and ultimately, life functions [2]. Moreover, concern exists regarding the expansion of these activities into previously undeveloped oceanic regions, including polar environments and coral reef habitats [3]. To assess the impacts of noise on marine fauna, the acoustic characteristics of the various noise sources must be quantified [2]. Underwater noise is measured using fixed or mobile hydrophones, including towed systems or sensors attached to marine animals, with data evaluated against environmental sound criteria [1], although commercial recorders can be costly and prone to loss or entanglement in fish nets [4].

Designing and constructing affordable alternatives from scratch can be challenging, as it requires extensive expertise in areas such as electronics design, embedded systems, and low-level programming [5]. The use of consumer-grade recorders [4] or microcomputer-based platforms, such as Raspberry Pi [5], provides a low-cost and efficient approach for underwater noise measurements, particularly in shallow coastal environments where high-pressure-resistant systems are not required.

A shallow-water autonomous underwater acoustic recorder was developed and implemented based on the Raspberry Pi 5 microcomputer platform. The system was designed to acquire and store acoustic data during mid-term deployment periods (up to approximately 30 days) while operating from rechargeable battery modules. The developed recorder extends the concepts and results previously presented in [5] and [6]. It was constructed using a commercially available pressure-resistant housing, a low-cost shallow-water hydrophone, additively manufactured structural components, and high-capacity LiFePO₄ battery modules. The developed device is suitable for various applications, including environmental underwater noise assessment, marine mammal monitoring, and the characterization of impulsive and vessel-generated noise in shallow-water environments.

The developed autonomous recorder provides a cost-effective alternative to commercially available underwater acoustic monitoring systems. The total material cost of the developed system was approximately €1350, which is considerably lower than that of comparable low-cost commercial solutions, such as the SoundTrap ST400 STD by Ocean Instruments (∼€3700), Sylence-LP by RTsys (∼€4000), and DSG Snap by Loggerhead Instruments (∼€4000). Despite its reduced cost, the developed system provides a flexible and customizable platform for mid-term shallow-water acoustic monitoring applications.

To validate system performance, the recorder was calibrated in air using a purpose-built acoustic coupler and sine wave signals spanning the one-third-octave frequency bands from 0.02 to 1 kHz. Calibration followed the comparative hydrophone calibration procedure outlined in [7]. Furthermore, an existing MATLAB® signal processing script for underwater noise acquisition [4] was adapted and optimized for use with the developed recording platform.

## Method details

The architecture of the developed system comprises the following main components: a hydrophone; a signal acquisition module equipped with an analog-to-digital converter (ADC); a power-supply unit with embedded power management electronics; a watertight enclosure; and firmware implemented as Raspberry Pi scripts.

The Aquarian Audio H2d rugged low-noise hydrophone was integrated into a Blue Robotics 6″ watertight enclosure designed for ROV/AUV systems. It was mounted on a 150 mm-diameter acrylic blank endcap using a 3D-printed coupling and epoxy potting to ensure mechanical stability and waterproofing. The H2d exhibits a sensitivity of −172 dB re 1 V/µPa, a usable frequency range of 0.01–100 kHz, and an omnidirectional horizontal polar response. Its physical dimensions are 25 × 46 mm, with a mass of 105 *g*. The H2dM variant provides a 3.5 mm TRS output requiring BIAS plug-in power for proper operation and is suitable for shallow water use up to 80 m depth.

The sound acquisition module was implemented using a Raspberry Pi 5 microcomputer paired with a HiFiBerry DAC+ ADC Pro high-resolution audio HAT. The Raspberry Pi 5 features a quad-core ARM Cortex-A76 processor clocked at 2.4 GHz and includes a built-in real-time clock (RTC). A micro-SD card slot is integrated into the board for data storage. The board measures 85 × 56 mm, weighs 46 *g*, and is powered via a 5 V USB connection.

The HiFiBerry DAC+ ADC Pro supports high-fidelity audio acquisition, with sample rates ranging from 44.1 to 192 kHz at 24-bit resolution. The ADC achieves a dynamic range of 110 dB, a maximum input voltage of 2.1 Vrms, and an adjustable input gain from −12 dB to +32 dB. The HAT measures 55 × 65 mm and has a mass of 30 *g*.

The power-supply unit was integrated into a 3D-printed tubular housing and comprised two 3.2 V LiFePO₄ rechargeable batteries with associated power management electronics, including a DC/DC converter (5–40 V input to 1.2–35 V output). During continuous recording, the supply current measured at the data acquisition board was 2.75 W. Endurance testing with a 2S LiFePO₄ battery pack demonstrated approximately 180 h (7.5 days) of continuous operation, corresponding to an available battery energy of ∼495 Wh. The detailed summary of the power consumption characteristics of the developed system can be found in Table 1.

**Table 1:** Measured power characteristics and derived battery capacity parameters of the recorder system.

Specification	Specified value
Battery configuration	2S LiFePO₄
Measured nominal battery voltage, $V_{bat}$	6.4 V
Measured recording board current and voltage, I;V	0.55 A @5 V
Measured operating time, t	180 h (7.5 days)
Recording power, P(P=VI)	2.75 W
Available energy, E(E=Pt)	495 Wh
Duty cycle, D	0.25 (15 min h⁻¹)
Average power demand, $P_{avg}\;$($P_{avg}=PD$)	0.69 W
Estimated battery capacity, C($C=E/V_{bat}$)	80 Ah⁎
Estimated autonomy, $t_{sc}\;$($t_{sc}=E/P_{avg}$)	717 h (29.9 days)

⁎ Values of battery energy and equivalent capacity were derived from laboratory endurance tests and should be considered experimental estimates rather than nominal battery ratings.

The Raspberry Pi-based firmware, implemented in Python, supported continuous and energy-saving recording modes with configurable gain and sampling rate. Low-power operation was achieved through audio-only recording with Wi-Fi and HDMI disabled. In energy-saving mode, the recorder was programmed to operate in a duty-cycled regime (15 min recording and 45 min low-power shutdown) controlled by an RTC-based scheduling mechanism. This reduced the average power consumption to ∼0.69 W and provided system autonomy of up to 30 days (720 h). It should be noted that autonomy estimates were derived from laboratory measurements at +20 °C and may vary under field conditions due to differences in system activity and environmental factors.

**Fig. 1:**
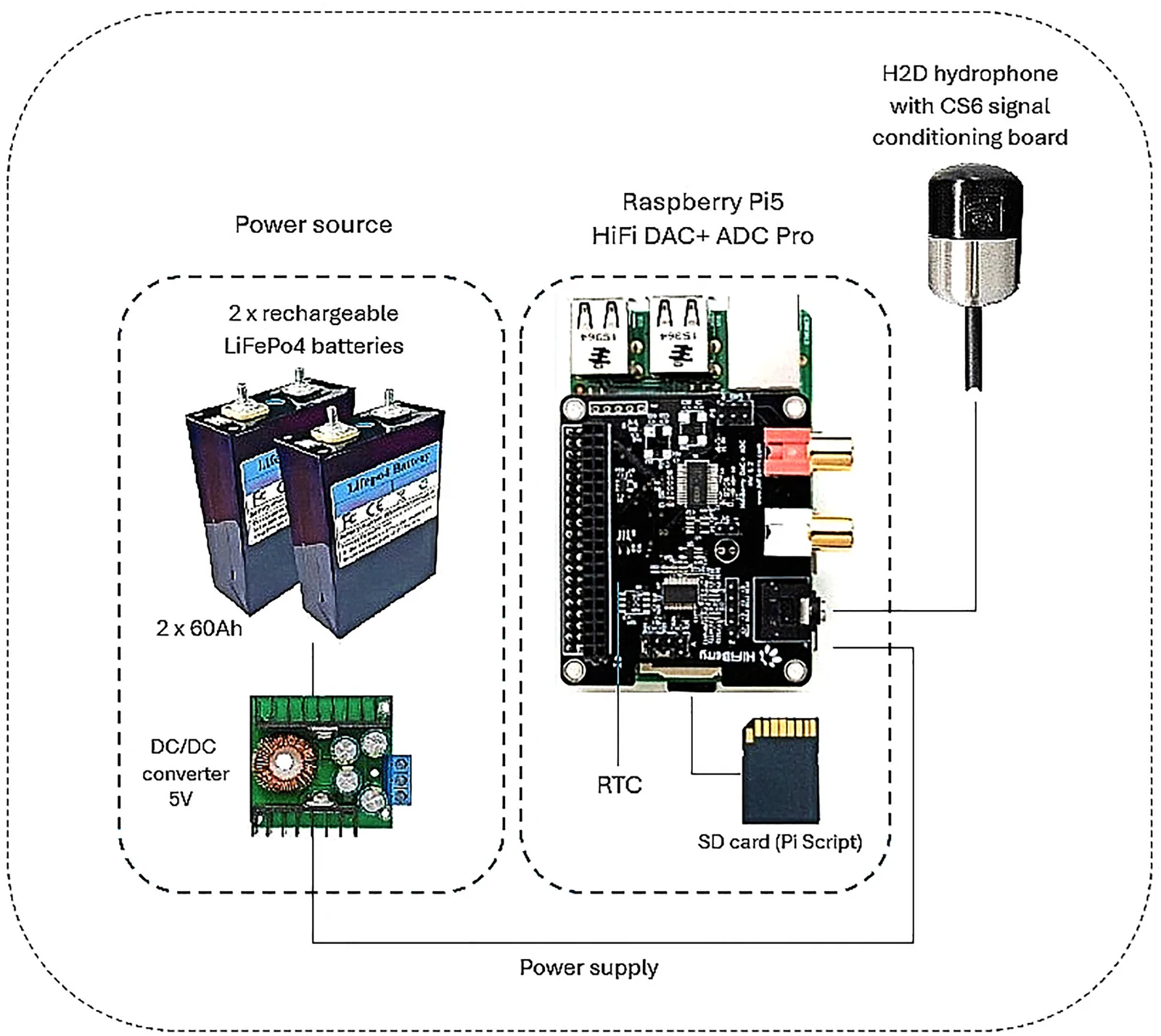
Schematics of the developed sound acquisition system.

All system components (Fig. 1) were housed in a modular watertight enclosure manufactured by Blue Robotics®, with an inner diameter of 152.4 mm and a length of 298 mm. The enclosure features alloy flanges, flat acrylic end caps, double O-ring seals, and an integrated pressure vent. It is designed for integration with ROVs, AUVs, and other marine applications and is rated for an operational depth of 65 m, making it suitable for coastal deployments. According to the manufacturer, the enclosure is produced under a documented quality assurance process that includes raw material inspection, in-process quality control, and end-of-line performance testing. Inspection and test records are linked to each unit's unique serial number, ensuring full product traceability.

For the intended underwater sound monitoring, a system’s gain setting of 13.2 dB and a sampling rate of 44.1 kHz were selected, with audio data segmented into 2-hour WAV files. All recordings are stored on a microSD card and named using the format <YYYYMMDD>_<HHMMSS>. During system testing, a real-time clock drift of 4 s per 24 h (0.167 s per hour) was observed in energy-saving mode; this drift should be accounted for during subsequent signal processing. The acquired WAV files were pre-processed and analyzed using WaveLab 7, followed by further processing using MATLAB software.

Although, the sound acquisition module incorporating the Raspberry Pi 5 with the HiFiBerry audio hat has limited autonomy for short to mid-term deployments, the module can be programmed to run real-time AI programs purposed for sound event detection, such as bioacoustics sounds and underwater noise classification including detection of time frequency patterns like whale calls or underwater machinery noise. This approach is feasible and actively researched in bioacoustics and underwater noise analysis [8]. On the other hand, to increase the autonomy of a device designed for underwater noise monitoring, the integration of an ESP32-family microcontroller sound board (i.e. ESP32‑S3 “Sonatino”) can be considered. These microcontrollers can extend the device’s operational time using the same battery capacity, although their ability to run real-time AI programs for sound processing is limited.

Finally, the developed underwater recording device was integrated into an alloy frame constructed from CNC modular structural rails with 20 × 20 mm profiles. The top and bottom covers were machined from 9 mm thickness marine plywood, resulting in overall alloy frame external dimensions of 330 × 205 × 205 mm.

## Method validation

Most anthropogenic underwater noise sources occur within the 0.02 – 1 kHz frequency range, making it essential to characterize the true frequency-dependent response of the measurement system across this acoustic spectrum. Furthermore, accurate determination of sound levels in the 63 Hz and 125 Hz 1/3-octave bands is particularly important, as these bands are among the key frequency bands considered for underwater noise assessment under the EU Marine Strategy Framework Directive (MSFD) guidelines. Therefore, the developed recording system was calibrated at the Klaipėda University Marine Research Institute on 18 June 2025, between 10:24 and 10:52 LT, at an ambient temperature of 22.8 °C, to determine the actual measurement response of the complete recording chain. The system was characterized by using sinusoidal signals at 1/3-octave center frequencies between 0.02 and 1 kHz, generated by a portable computer and an online tone generator [9]. The calibration followed the methodology developed within the EMPIR UNAC-LOW (Underwater Acoustic Calibration Standards for Frequencies Below 1 kHz) project, which employs an air-filled coupling chamber to provide traceable calibration of hydrophones and autonomous underwater acoustic recorders at low frequencies. The methodology is based on existing secondary hydrophone calibration procedures specified in the International Electrotechnical Commission standard IEC 60,565:2006 and is intended for environmental underwater noise measurements [10].

**Fig. 2:**
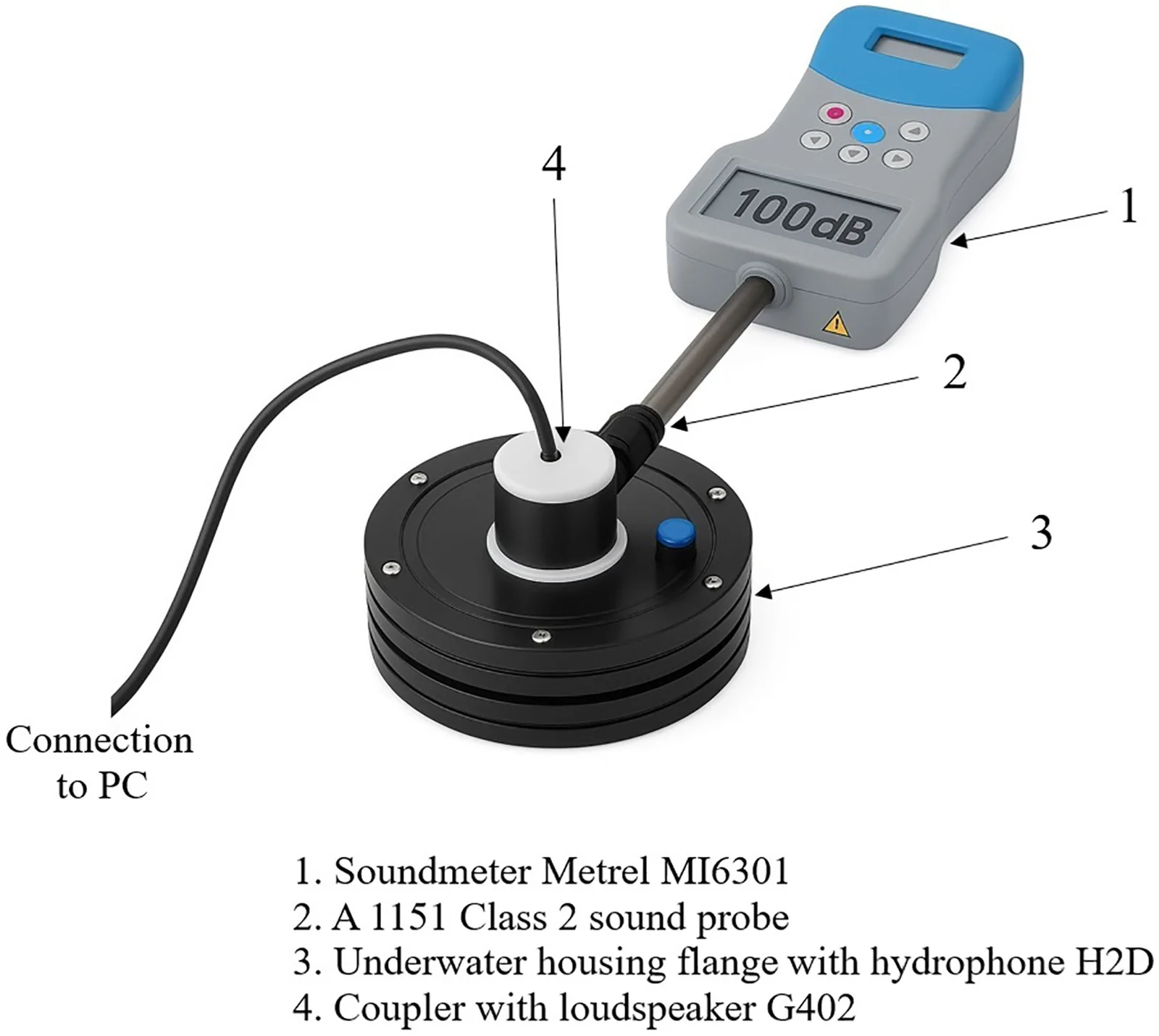
Calibration setup used for the hydrophone system. The calibration chamber features an internal diameter of 26 mm for the hydrophone under test and 11 mm for the sound probe, with length of 30 mm. Image created with ChatGPT (OpenAI, 2026).

A coupling chamber for calibration of the developed sound acquisition system was designed following the methodology described by [11,12]. The calibrator chamber incorporated a G402 miniature loudspeaker with a flat frequency response over the range of 0.02–20 kHz. The hydrophone and a calibrated reference sound-level meter probe A1511 were placed inside the air-filled chamber in close proximity, ensuring that both sensors were exposed to the same acoustic pressure. The reference sound-level meter Metrel DUS Fons MI 6301 (S/N 13,190,027) is accompanied by manufacturer-provided calibration data. Factory calibration was performed using a Brüel & Kjær multifunction acoustic calibrator (S/N 2295,558), and the complete system holds an ISO calibration certificate (No. PS3–14–002). The developed calibration setup is shown in Fig. 2.

**Fig. 3:**
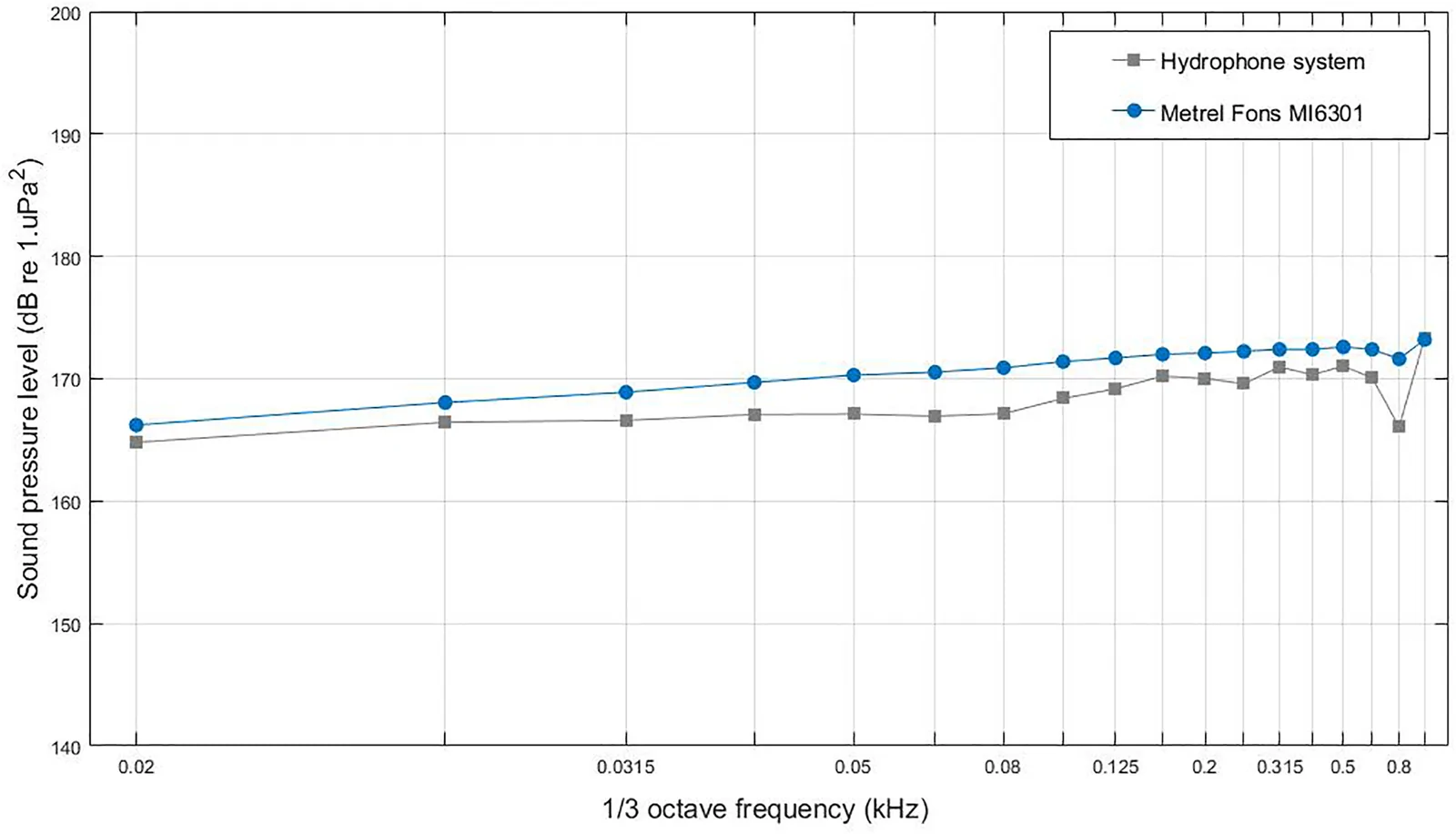
Comparison of the sound pressure levels, acquired in calibration chamber.

The comparison results of the measured true levels of the sine-wave signals in 1/3-octave center bands over the frequency range of 0.02–1 kHz, before applying the calibration constants, are shown in Fig. 3.

The test results demonstrate a relatively flat frequency response over the 0.02–0.7 kHz range. However, increased sound pressure variations were observed in the 0.8–1 kHz range, as detected by both the developed sound acquisition system and the reference sound level meter. These deviations indicate limitations of the applied coupler design at higher frequencies. In particular, the use of a single reference microphone and the internal spacing between the microphone diaphragm and the hydrophone surface may introduce acoustic effects that limit the achievable upper calibration frequency, resulting in reduced accuracy near the intended 1 kHz limit [11]. This limitation can be mitigated by using a liquid-filled coupling chamber, where the longer acoustic wavelength compared with gas reduces the influence of chamber dimensions and enables extension of the usable calibration frequency range [10].

The self-noise of the designed system was tested on the premises of the Klaipėda Higher Education Institution (KVK) during the nighttime. The measured noise levels were compared with the theoretical zero-level noise of the Baltic Sea, computed using the wind-dependent spectrum model [13], expressed as follows (equation No.1):

(1) $$NSL\left(f,U\right)=max\left\{ \begin{array}{c} 24Log_{10}\left(1+U\right)-17\left(f\right)+35\\ 20Log_{10}\left(f\;/\;6\right)\; \end{array}\right.$$

Here *U* is the wind speed in knots, *f* is the frequency in kHz, max denotes the greater of the two values considered, and *NSL* is the noise spectrum level in dB re 1 μPa²/Hz.

**Fig. 4:**
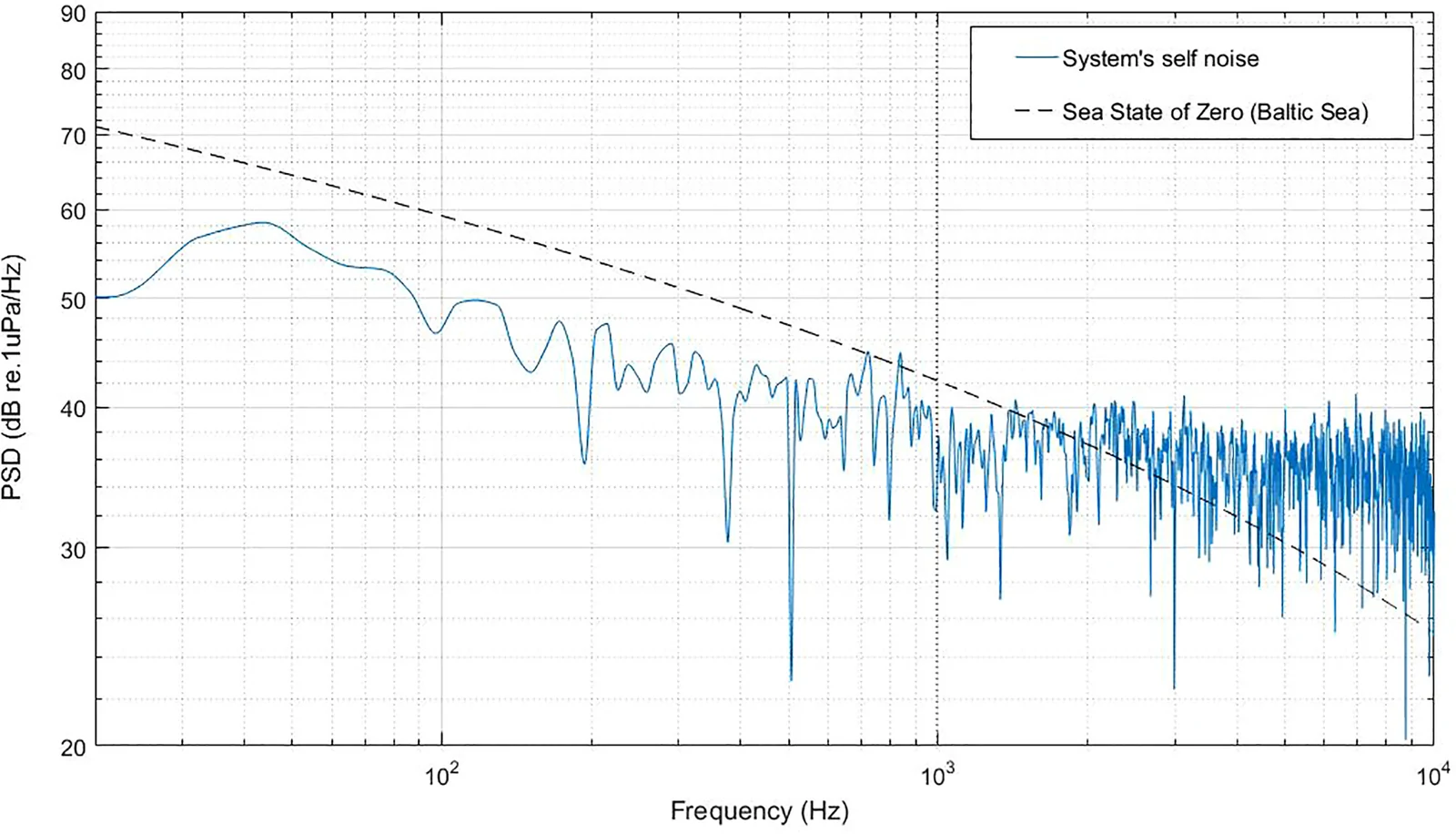
System’s self-noise levels compared with the theoretical zero-sound-level sea state.

The comparison results of the acquired system’s self-noise spectrum levels, along with the Sea state of zero sound levels, are shown in Fig. 4.

The sensitivity of the sound recording device at its fundamental frequency was quantified as the ratio of its output voltage to the ambient sound pressure, as expressed by the equation No.2 [14]:

(2) $$M_{v}=\frac{V_{h}}{P_{h}}$$

Where *V_h_* is the read out of the voltage and pH is the pressure in μPa. The amplitude voltage signal produced by the system *V_h_*, was determined by recording the system's amplitude values using the equation No.3 [15]:

(3) $$V_{h}=\frac{V_{peak}X_{bits}}{2^{N-1}}=V_{peak}X_{wav}$$

Where the *V_peak_* is the zero-to-peak voltage range of the ADC and $X_{bits}=2^{N-1}X_{wav}\;$(with N being the bit resolution of 24). The sensitivity of the recording chain reached −191.96 dB re 1 V/μPa in the 1 kHz band, compared to the hydrophone sensitivity of −172.0 dB re 1 V/μPa.

The key characteristics of underwater sound recording systems - sensitivity, self-noise, dynamic range, directional response, and frequency response must be evaluated within the frequency bands of interest [7]. The specifications of the developed sound acquisition system are summarized in Table 2.

**Table 2:** Key characteristics of the developed underwater sound acquisition system.

Specification	Specified value
Hidrophone sensitivity (wideband)	−172 dB re 1V/µPa
Reording system’s sensitivity (1 kHz)	−192 dB re1V/µPa
Self noise (1 kHz)	37 dB re 1µPa/Hz
Dynamic range (ADC)	110 dB
Directional response	Omnidirectional (horizontal)
Frequency responce	0.01 – 96 kHz (0.01 – 70 kHz⁎)
Total harmonic distortion	−90 dB (∼0.003%)
Depth rating	65 m

⁎ Limited by the Hifiberry hat (as provided by the manufacturer).

**Fig. 5:**
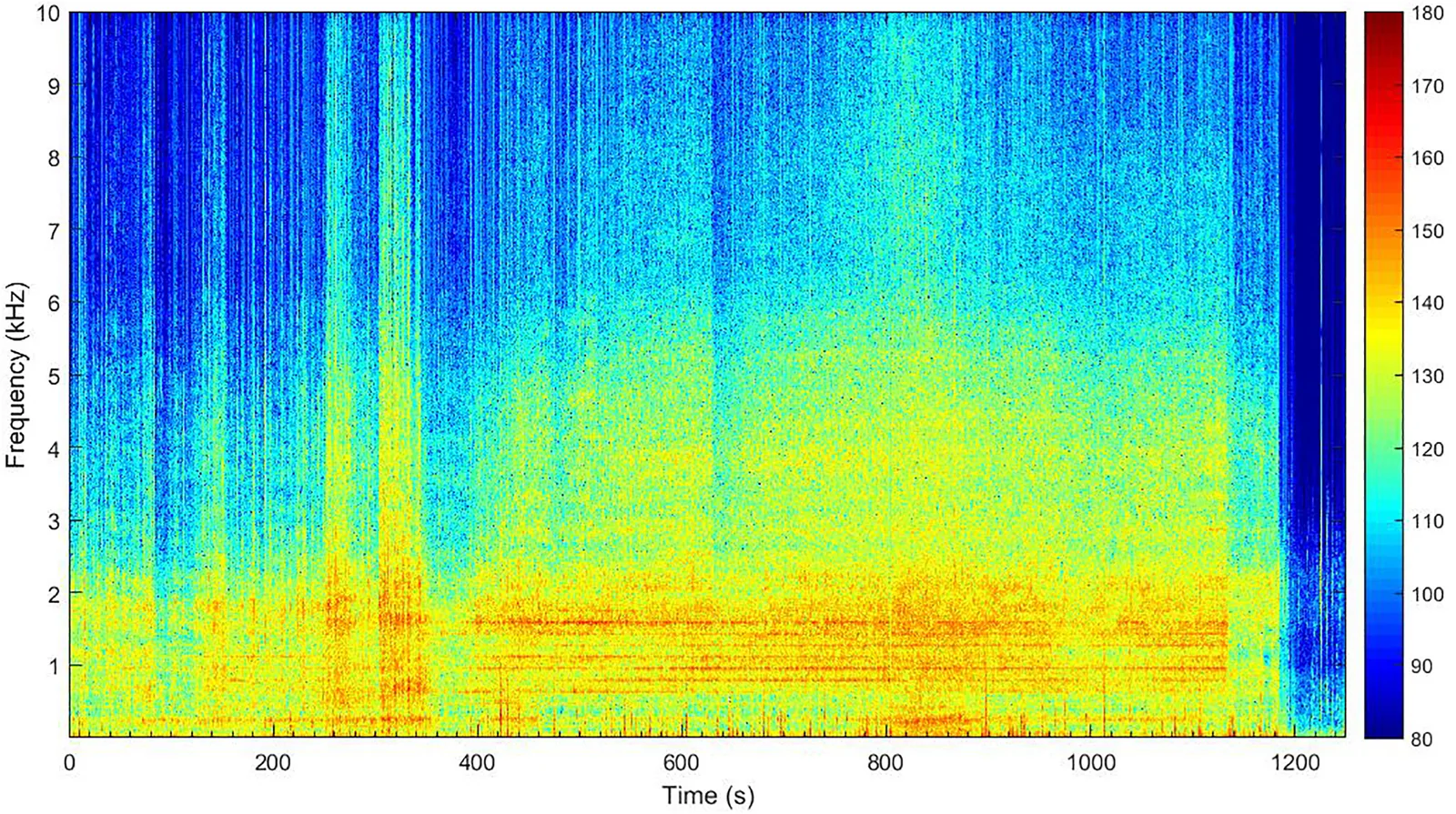
Spectrogram of the measured underwater sound generated by a passing containership (LOA 237 m) at the speed of 8.8 knots, recorded at distance of 250 m (color bar represents the units dB re 1 µPa^2^/Hz**)**.

To test the developed prototype, a mini field research campaign was conducted on 2026–05–01, during which the underwater recording setup was submerged from the berth to a depth of 1.5 m in the Klaipėda Harbour area (location N55.687,485°, E21.129,251°). During the experiment, the underwater noise signature of a containership with a length overall (LOA) of 237 m was recorded (CPA on 10:28LT) while it was navigating at a speed of 8.8 knots at its closest point of approach (CPA) equal to ∼250 m (ships data retrieved from [16]). The resulting ship-radiated noise spectrogram is shown in Fig. 5. The recorded acoustic data were processed using underwater sound signal-processing script employing a Fast Fourier Transform (FFT) with a Hanning window, a 4096-point window size, and a 1-second time resolution.

## Limitations

The prototype underwater sound acquisition system currently has two main limitations: a maximum depth rating of 65 m and limited operational autonomy. These can be addressed by using a Blue Robotics 500 mm Locking-Series aluminum enclosure rated for depths up to 1000 m, as well as installing an Aquarian Audio S1-series hydrophone, which is rated to 200 m, making the system suitable for continental shelf applications. Device autonomy can be further improved by adding additional LiFePO₄ battery cells and replacing the signal acquisition board with ESP32-family microcontroller electronics.

## CRediT author statement

Donatas Bagočius: Conceptualization, Methodology, Data curation, Signal Processing, Writing - Original draft preparation. Aleksas Narščius: Conceptualization, Data curation, Programming, Signal Processing, Technical assistance. Gintaras Kučinskas: Technical drawings, 3D printing, technical assistance. Gediminas Uskovas: Firmware – Pi script writing, Technical assistance.

## Declaration of generative AI and AI-assisted technologies in the manuscript preparation process

During the preparation of this work the authors used OpenAI. (2026) to optimize the MATLAB signal processing script and generate Fig. 2 based on provided photo (AI-generated image). After using this tool, the authors reviewed and edited the content as needed and takes full responsibility for the content of the published article.

## Declaration of competing interest

The authors declare that they have no known competing financial interests or personal relationships that could have appeared to influence the work reported in this paper.

## Data Availability

Data will be made available on request.

## Appendix Supplementary materials

Image, application 1
