# Supplementary material for: Development of a shallow-water underwater acoustic recording system — version 2
Source: MethodsX. 2026 Aug 22;17:104112. doi: 10.1016/j.mex.2026.104112 (PMC13544172; doi:10.1016/j.mex.2026.104112)
Supplement: Supplementary file 1 [file mmc1.zip › DEVICE CALIBRATION DATA SHEET.pdf]

DEVICE CALIBRATION DATA SHEET

Document Reference: CAL-001

Date of Issue: June 18, 2025

Execution Location: Marine Research Institute, Klaipeda University

---

1. ENVIRONMENT & TEST CONDITIONS

- **Calibration Date:** June 18, 2025
- **Time Period:** 10:24 – 10:52 LT
- **Ambient Temperature:** +22.8 °C
- **Status:** Supplementary Calibration Data

---

2. CALIBRATION SPECIFICATIONS & METHODOLOGY

- **Target System:** acoustic recording system V2.
- **Input Signal Type:** Narrow-band sinusoidal signals.
- **Frequency Standard:** One-third-octave centre frequencies.
- **Test Range:** 20 Hz to 1 kHz.
- **Statistical Basis:** Five (5) repeated measurement cycles per center frequency.
- **Data Synthesis:** Arithmetic mean (Average) and sample standard deviation (σ) derived from 5 test iterations.

---

3. CALIBRATION DATA

All values are expressed in decibels (dB) re 1 μPa^2.

Frequency (Hz)	Hydrophone Mean	Hydrophone Std Dev (σ)	Noise Meter Mean	Noise Meter Std Dev (σ)	Delta (Δ)
**20 Hz**	164.81	0.267	166.22	0.110	1.41
**25 Hz**	166.46	0.081	168.06	0.055	1.61
**31.5 Hz**	166.60	0.040	168.90	0.000	2.30
**40 Hz**	167.08	0.049	169.70	0.000	2.62
**50 Hz**	167.11	0.028	170.30	0.000	3.19
**63 Hz**	166.95	0.005	170.54	0.018	3.60
**80 Hz**	167.14	0.004	170.90	0.000	3.76
**100 Hz**	168.39	0.008	171.40	0.000	3.01
**125 Hz**	169.18	0.055	171.70	0.000	2.52
**160 Hz**	170.21	0.005	172.00	0.000	1.79
**200 Hz**	170.03	0.000	172.10	0.000	2.07
**250 Hz**	169.58	0.009	172.24	0.084	2.66
**315 Hz**	170.97	0.013	172.40	0.000	1.44
**400 Hz**	170.33	0.011	172.40	0.000	2.08
**500 Hz**	171.03	0.005	172.44	0.089	1.57
**630 Hz**	170.07	0.000	172.40	0.000	2.33
**800 Hz**	166.07	0.008	171.62	0.045	5.55
**1000 Hz**	173.31	0.004	173.20	0.000	-0.11

---

4. CALIBRATION TOLERANCE

- **Instrument Stability (Hydrophone recording system):** High baseline consistency is observed at mid frequencies, peaking in stability at **200 Hz** and **630 Hz** (σ = 0.000). The maximum variance is noted at **20 Hz** (σ = 0.267).
- **Instrument Stability (Noise Meter):** The Noise Level Meter shows absolute quantization lock (σ = 0.000) across 12 of the 18 bands. Minor variance peaks appear at **250 Hz** (σ = 0.084) and **500 Hz** (σ = 0.089).
- **Systematic Deviation:** The reference Noise Level Meter shows systematically higher response values relative to the Hydrophone Recorder across 94% of evaluated center frequencies. A maximum delta (Δ) of **5.55 dB** is identified at the **800 Hz** tracking node.
