# Supplementary material for: Development of a shallow-water underwater acoustic recording system — version 2
Source: MethodsX. 2026 Aug 22;17:104112. doi: 10.1016/j.mex.2026.104112 (PMC13544172; doi:10.1016/j.mex.2026.104112)
Supplement: Supplementary file 1 [file mmc1.zip › METREL DUS Calibration sertifcate.pdf]

METREL® DUS

Calibration Laboratory

http://www.metrel.si
E-mail:metrel@metrel.si

METREL DUS d.d.
Ljubljanska cesta 77
1354 Horjul, Slovenia
Phone: +3861 7558 200
Fax: +3861 7549 095
VAT No.: SI 78 997 933

CALIBRATION CERTIFICATE

Number: **PS3-14-002**

Customer	
Instrument	**Fons**
Type	**MI 6301**
Serial No.	**Instrument: 13190027**
Manufacturer	**Metrel**
Calibration procedure	Calibration was carried out by comparison of values indicated, or set, on the item under calibration, with values of measurands, realized with measurement standards. Detailed descriptions, where necessary, are given along with measurement results.
Environmental conditions	Temperature: 23 °C ± 5 °C Relative humidity: 20 % to 70 %
Date of calibration	**12.3.2014**
Measurement results	are given on the following pages
Measurement uncertainty	The measurements have been executed using standards which the traceability to (inter)national standard is guaranteed. The reported expanded uncertainty of measurement is stated as the standard uncertainty of measurement multiplied by coverage factor k=2. which for a nominal distribution corresponds to a coverage probability of approximately 95%. The standard uncertainty of measurement has been determined in accordance with EA Publication EA-4/02.

Performed by:	*Approved by:* *Head of the Calibration Laboratory*	*Date of issue:*
**Jernej Sedej**	**Janko Mole**	**12.3.2014**

METREL DUS
družba za upravljanje in svetovanje d.d.
Ljubljanska cesta 77
1354 Horjul
1

Reproduction of the complete certificate is allowed. Parts of the certificate may only be reproduced with written approval of the calibration laboratory.

CALIBRATION CERTIFICATE

Calibrated parameters:

1. Sound pressure level

Reference equipment:

	Serial No.
Multifunction Acoustic Calibrator, 4226, Bruel_Kjaer	2295558

Calibration procedure:

Sound pressure level was calibrated with multifunction acoustic calibrator.

Abbreviations and symbols:

Parameters marked with index "s" represent the ("true") value of the measurand
Parameters marked with index "x" represent values indicated or set on the calibrated item

E = X – S	error of indication of the item under calibration
e = (X – S) / S	relative error of indication of the item under calibration
(me), mE	(relative) limit of error, specification
(n), N	(relative) expanded measurement uncertainty, expressed as standard uncertainty, multiplied by the coverage factor, k=2

Limit of error for individual measured parameters are stated along with the measurement results are calculated from manufacturer's specifications given in manuals for:

MI 6301, Ver 1.0, Code No. 20750068
A 1151, Ver 1.0, Code No. 20750882

CALIBRATION CERTIFICATE

Measurand		s	x	N	E	mE

Sound probe **A1151, Class2** Serial number **13190129** Mic ser. No.: 503108

Sound pressure level

Range	Frequency	dB	dB	dB	dB	dB
30 dB - 110 dB	31,5 Hz	93,95	93,7	0,2	-0,3	3,5
30 dB - 110 dB	63 Hz	93,99	93,8	0,2	-0,2	2,5
30 dB - 110 dB	125 Hz	94,00	93,8	0,2	-0,2	2,0
30 dB - 110 dB	250 Hz	94,04	93,9	0,2	-0,1	1,9
30 dB - 110 dB	500 Hz	94,05	93,8	0,2	-0,2	1,9
30 dB - 110 dB	1000 Hz	94,0	94,0	0,2	0,0	1,4
30 dB - 110 dB	2000 Hz	94,03	94,0	0,2	0,0	2,6
30 dB - 110 dB	4000 Hz	94,0	94,5	0,2	0,5	3,6
30 dB - 110 dB	8000 Hz	94,0	96,4	0,2	2,3	5,6
40 dB - 120 dB	1000 Hz	94,04	94,0	0,2	0,0	1,4
40 dB - 120 dB	1000 Hz	114,0	114,0	0,2	0,0	1,4
50 dB - 130 dB	1000 Hz	114,0	114,0	0,2	0,0	1,4
60 dB - 140 dB	1000 Hz	114,0	114,0	0,2	0,0	1,4
